# Stent-Assisted Coiling versus Coiling in Treatment of Intracranial Aneurysm: A Systematic Review and Meta-Analysis

**DOI:** 10.1371/journal.pone.0082311

**Published:** 2014-01-15

**Authors:** Yuan Hong, Yong-Jie Wang, Zheng Deng, Qun Wu, Jian-Min Zhang

**Affiliations:** Department of Neurosurgery, Second Affiliated Hospital, School of Medicine, Zhejiang University, Hangzhou, Zhejiang Province, China; National Taiwan University, Taiwan

## Abstract

**Background and Purpose:**

Stent-assisted coiling was initially invented for wide-neck aneurysms, but is now used for smaller berry aneurysms. The aim of this study was to compare the safety and efficiency of stent-assisted coiling with conventional coiling in treatment of intracranial aneurysms.

**Methods:**

A meta-analysis of studies that compared stent-assisted coiling with coiling only was conducted by searching English literatures via Pubmed, Medline and Cochrane Library databases without restricting the publication year. The primary outcomes in this study were immediate occlusion, progressive thrombosis rate, all-complication rate and angiographic recurrence. The secondary outcomes examined were packing density, mortality, permanent complication and thromboembolic complication rate.

**Results:**

Ten retrospective cohort studies were included. There is currently only one unfinished randomized study. Although the stent-assisted coiling group tended to show a lower initial occlusion rate than that of the coiling-only group (57.6% versus 68.7%; OR, 0.66; 95% CI, 0.30–1.44; P = 0.30), it achieved a significantly higher progressive thrombosis rate during follow up compared to that of the coiling only group (37.5% versus 19.4%; OR, 2.75; 95% CI, 1.95–3.86; P<0.00001) and a significantly lower recurrence rate (16.2% versus 34.4%; OR, 0.35; 95% CI, 0.25–0.49; P<0.00001). With respect to safety concerns, the all-complication rate (17.6% versus 15.9%; OR, 1.12; 95% CI, 0.77–1.62; P = 0.56), mortality rate (9.1% versus 2.6%; OR, 2.31; 95% CI, 0.68–7.82; P = 0.18), permanent complication rate (5.6% versus 3.9%; OR, 1.52; 95% CI, 0.96–2.41; P = 0.08) and thromboembolic complication rate (4.2% versus 4.9%; OR, 0.99; 95% CI, 0.41–2.38; P = 0.97) did not show significant difference between the two groups.

**Conclusions:**

Stent-assisted coiling has a lower recurrence rate than conventional coiling. Analysis of complication events did not show any significant difference between the two methods. Despite the findings reported herein, further validation by well-designed prospective studies is needed.

## Introduction

Since the first introduction of detachable coiling in 1990, it has been widely applied for the treatment of intracranial aneurysms [1]. However, recanalization is a problem of aneurysmal coiling, limiting its use to wide-neck aneurysms [2]. Alternatively, stent-assisted coiling was invented, initially for wide-neck aneurysms, based on the hypothesis that a stent can provide the scaffold to hold the coils in the aneurysmal cavity [3], [4], [5]. More recently, stent-assisted coiling has been used to treat smaller berry aneurysms [6]. Future technological developments will likely focus on improving the durability of endovascular treatment by delivering bioactive agents that promote aneurysm thrombosis rather than only the coil [7].

Until now, the safety issues and efficiency of stent assisted coiling have not been fully evaluated. Several cohort studies comparing the stent-assisted coiling and coiling only techniques have been published, however the results were controversial [6], [8], [9], [10], [11], [12], [13], [14], [15], [16]. Two systematic reviews concluded that stent-assisted coiling seemed to have more adverse events than traditional coiling [17], [18], but these studies were limited since only data on stent-assisted coiling was used. Here, we present the results of a systematic review and meta-analysis to evaluate the aneurysmal occlusion, recurrence and complication occurrence between two different endovascular techniques: stent-assisted coiling and conventional coiling.

## Methods

### Systematic literature search

English publications comparing stent-assisted coiling and coiling only for patients with cerebral aneurysm were searched using Pubmed, Medline and Cochrane Library databases without restricting the year of publication. Title, abstract, keywords and free text were searched using combinations of the following keywords: intracranial or cerebral or carotid or basilar, aneurysm or aneurysms, stent, coil, patient. The identified articles were reviewed by two authors (Yuan Hong and Yongjie Wang) independently. In addition, identified articles were also reviewed for other studies. For those studies that generated multiple publications, the most relevant data was extracted for analysis.

### Inclusion and exclusion criteria

The criteria for inclusion of a study were those that: (1) compared stent-assisted coiling and coil­ing only; (2) reported patients who had definite intracranial aneurysms, whether ruptured or not, verified by computed tomography, magnetic resonance imaging or angiography; (3) reported occlusion rate, complications, clinical outcomes or angiographic recurrence re­ported for both groups; (4) included treatment of at least 5 aneurysms for each group. Since it’s difficult to randomly assign the patients to stent-assisted coiling or coiling only groups, regardless of the morphological features and rupture status of aneurysms [9], cohort studies with substantial imbalance of related clinical characteristics were still included.

The exclusion criteria were the studies that: (1) reported patients who had dissecting aneurysms; (2) reported patients who received treatment other than stent-assisted coiling and coiling only; (3) had insufficient baseline information; (4) had insufficient data or made no comparison between the two target groups. In addition, all editorials, letters, review ar­ticles, case reports, and animal experimental studies were excluded.

### Selection and data extraction

The decision on whether a study should be included was made in­dependently by two authors (Yuan Hong and Yongjie Wang), with disagreements settled by the senior authors (Qun Wu and JianMin Zhang).

The primary outcomes were immediate occlusion and progressive thrombosis rate, all-complication rate and angiographic recurrence. In calculating occlusion rate, most articles adopted either the Raymond-Roy classification, defined as no filling, residual filling of the neck or residual filling of the neck and dome [19], or the modified quantitative scheme described by Kole et al., defined as complete obliteration with 100% occlusion, near-complete with greater than 95% occlusion and incomplete obliteration with less than 95% occlusion [20]. It is well known that partial occlusion below 95% may lead to more unfavorable results [20]. In order to include as many useful data as possible, both no residual filling and occlusion rate >95% were counted for occlusion rate. Progressive thrombosis was defined as any increase of packing density on follow up angiography. While recurrence was defined as a decreasing extent of occlusion on follow up angiography. The secondary outcomes included packing density, mortality, permanent complication and thromboembolic complication.

### Quality assessment and statistical analysis

The bias of cohort studies was evaluated with the Newcastle-Ottawa scale. Meta-analysis was performed using the soft­ware package RevMan5.0. Dichotomous variables were presented as odds ratios (OR; stent-assisted coiling versus coiling only) with a 95% confidence interval (CI). Continuous variables were presented as mean values. Statistical heterogeneity was assessed using the I^2^. In cases of I^2^ larger than 50%, a random-effect model was used. Otherwise a fixed-effect model was used. Significance was set at P = 0.05. Funnel plots were used to screen for potential publication bias. Although the prognosis of ruptured and unruptured intracranial aneurysms was different, subgroup analysis was not conducted because, except for one report that presented data solely on ruptured aneurysms [15] and one on unruptured aneurysms [11], no data could be extracted separately from other included studies.

## Results

**Figure 1:**
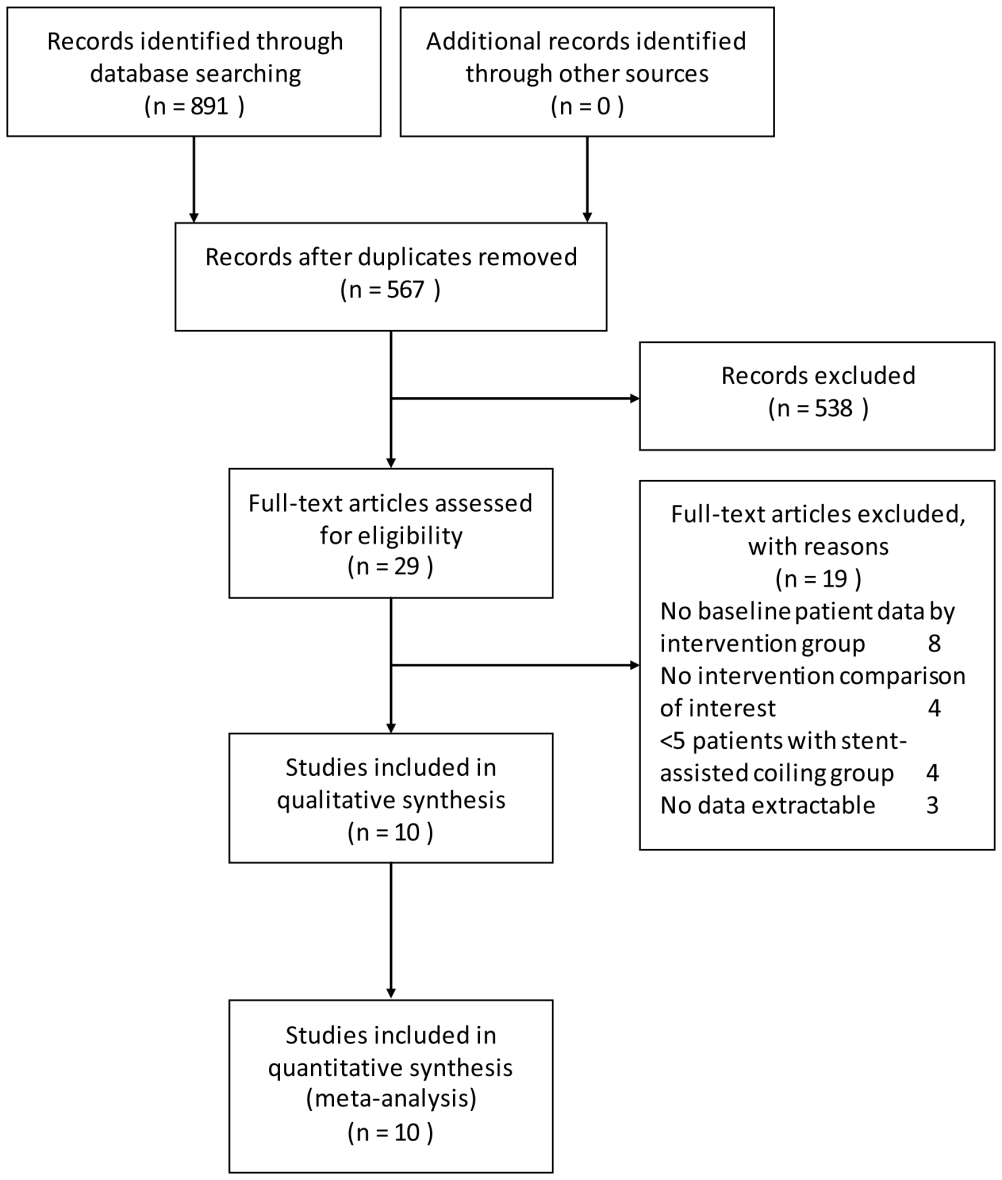
Flowchart of studies to final number of eligible studies.

Our search strategy revealed a total number of 567 different articles, 538 of which were excluded by title and abstract screening. Of the 29 articles left, full texts were accessed and 10 articles fulfilling the inclusion criteria were included [6], [8], [9], [10], [11], [12], [13], [14], [15], [16]. 19 of 29 articles were excluded because of either no baseline patient data (n = 8), no intervention comparison was made between stent-assisted coiling and coiling only (n = 4), less than 5 patients within interventional groups (n = 4) or no extractable data (n = 3). Figure 1 shows a flow chart illustrating the above searching process.

### Baseline characteristics of included studies

All the 10 articles included were retrospective cohort studies. Until now, only one randomized study of endovascular treatment of unruptured intracranial aneurysms with or without stents is still an ongoing clinical trial and no data is extractable [21]. The study by Ogilvy et al. was a subgroup analysis of the study by Jahshan et al., but it was still included because it contained extra data on aneurysm size and thromboembolic complications. The baseline characteristics of the included studies are summarized in table 1. In total, these studies included 2566 patients of whom 753 underwent stent-assisted coiling, and 1813 underwent endo­vascular coiling only. The size of aneurysms receiving stent-assisted coiling ranged from 6.6 mm to 11.5 mm, with a mean value of 8.6 mm, compared to the coiling only group range from 7.0 mm to 9.7 mm, with a mean value of 7.3 mm. The baseline characteristics of 2 studies showed significantly larger aneurysm size in the stent-assisted group [6], [13]. The percentage of ruptured aneurysms was 51.4% in the coiling only group compared with 24.0% in the stent-assisted group. 3 studies had significantly higher rate of aneurysm rupture in the coiling only group compared to that in the stent-assisted coiling group [6], [9], [13].

**Table 1:** Design and baseline characteristics of included trials.

First Author	Aneurysm	Age	Male	Size	Ruptured	Hunt & Hess		Follow up
	No.	y	%	mm	%	I-III		mo
	stent-assisted/coiling without a stent							
Albuquerque [8] 2011	8/9	52/57	27/25	NA	0/44	NA		12/7
Chalouhi[9] 2012	88/147	57/54	22/26	8.0/7.9	40/60	65.7/67.4		17/27
Colby[10] 2013	30/60	53/52	10/8	7.0/8.8	7/20	100.0/83.3		14/38
Gordhan[11] 2011	25/12	61/64	20/42	11.5/8.1	0/0	0/0		17/16
Izar[12]2011	84/84	56/56	11/11	7.5/7.5	8/14	NA		19/NA
Jahshan[13] 2013	225/264	category^a^	23/31	category^a^	20/62	78.3/70.9		18
Kim[14] 2010	37/37	59/55	19/35	6.6/7.0	22/22	NA		14/18
Kung[15] 2011	40/91	62/61	52/29	NA	100/100	62.5/60.4		NA
Ogilvy[16] 2011	70/24	54/56	14/0	10.4/9.7	11/8	NA		30/29
Piotin[6] 2010	216/1109	51/50	24/33	9.3/7.1	16/50		NA	14/22

No., number; y, year; mm, millimeter; mo, month; NA, not available.

a , the data were presented in a categorized manner.

### Technical approaches

For stenting in unruptured aneurysms, systemic anticoagulation with heparin was used to achieve an activated clotting time at more than twice or 250s, and dual antiplatelet therapy was administrated preoperatively for several days, and continued for at least 3 weeks in all the included studies. Chalouhi et al. and Jahshan et al. tested patients’ responses to both aspirin and clopidogrel, and, for non-responders, other effective drugs, such as prasugrel or ticlopidine, were applied [9], [13].

For stenting in ruptured aneurysms, a preoperative loading dose of antiplatelet medication was regularly administrated, and heparin was used after deployment of the coil [9], [10], [12].

A number of different stents were included in this meta-analysis; Neuroform (402 of 598 patients, 67.2%) and Enterprise self-expanding stents (130 of 598 patients, 21.7%) were the most commonly used stents, while 9 patients received both types [6], [8], [9], [10], [11], [12], [14], [15], [16]. Various stent techniques were conducted, including balloon remodeling, stent after coiling, stent before coiling, bail-out stent, stent-jack, jailed catheter and Y-configuration stent [6], [9], [11].

### Quality assessment

We evaluated the risk of bias for all 10 included studies using a Newcastle-Ottawa scale as displayed in table 2. In evaluating the quality of outcomes for each study, the follow up period was set at 1 year and follow up rate was selected to be 80%. The subjects lost to follow up were not adequately described in a majority of studies.

**Table 2:** Risk of bias in the observational studies using Ottawa-Newcastle rules.

	Selection	Comparability	Outcome
Albuquerque[8] 2011	★★★		★★
Chalouhi[9] 2012	★★★	★★	★★
Colby[10] 2013	★★★	★★	★★
Gordhan[11] 2011	★★★	★★	★★
Izar[12] 2011	★★★	★★	★★
Jahshan[13] 2013	★★★★	★★	★★★
Kim[14] 2010	★★	★★	★★★
Kung[15] 2011	★★★★	★★	★★
Ogilvy[16] 2011	★★★	★★	★★★
Piotin[6] 2010	★★★★	★★	★★★

Follow up period set as 1 year. Follow up rate set as 80%.

### Analysis of primary outcomes

**Figure 2:**
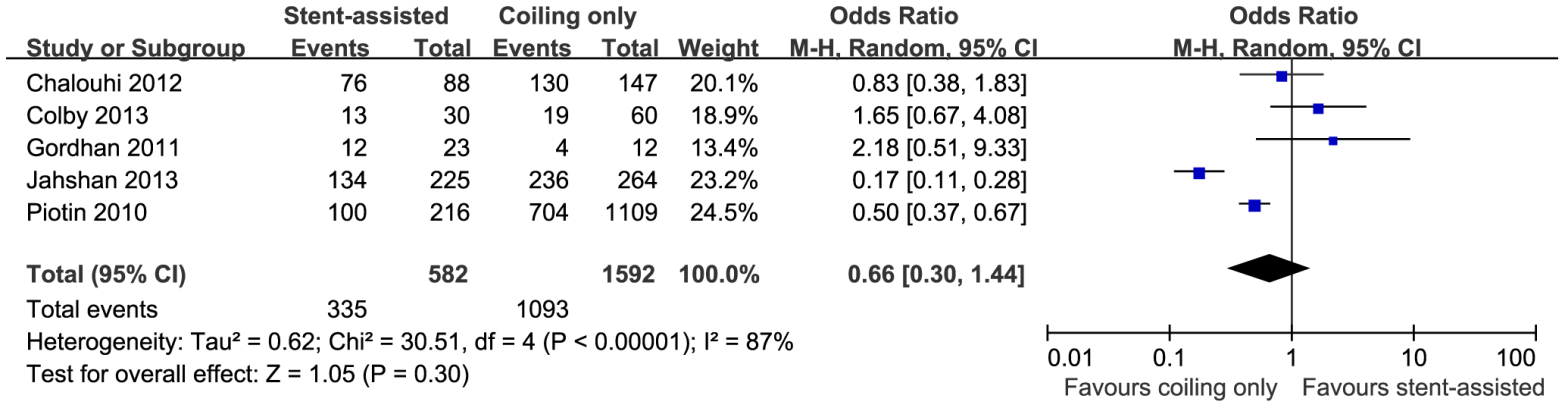
Forest plot of immediate occlusion rate comparing stent-assisted coiling versus coiling only. Random-effect model was applied.

**Figure 3:**
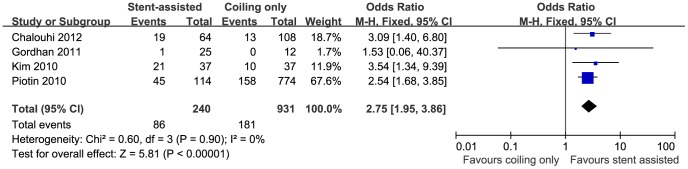
Forest plot of progressive thrombosis comparing stent-assisted coiling versus coiling only. Fixed-effect model was applied.

5 studies [6], [8], [9], [10], [11], [13] with a total number of 2174 patients reported the immediate occlusion rate after interventional treatment (582 patients received stent-assisted coiling and 1592 coiling only). In a fixed-effect model, the immediate occlusion rate of stent-assisted group was significantly lower than that of coiling only group (57.6% versus 68.7%; OR, 0.45; 95% CI, 0.36–0.56; P<0.00001) (Figure S1). When I^2^ was larger than 50%, a random-effect model was used, which only displayed tendency towards higher occlusion rate after treatment with conventional coiling compared to stent-assisted coiling (57.6% versus 68.7%; OR, 0.66; 95% CI, 0.30–1.44; P = 0.30) (Figure 2). 4 studies [6], [9], [11], [14], with a total number of 1171 patients, further analyzed the progressive thrombosis during follow up (240 patients received stent-assisted coiling and 931 coiling only). In contrast to the immediate occlusion rate, the stent-assisted coiling group showed a significant angiographic improvement during follow up compared to coiling only (37.5% versus 19.4%; OR, 2.75; 95% CI, 1.95–3.86; P<0.00001) (Figure 3). The heterogeneity analysis was insignificant (I^2^ = 0%).

**Figure 4:**
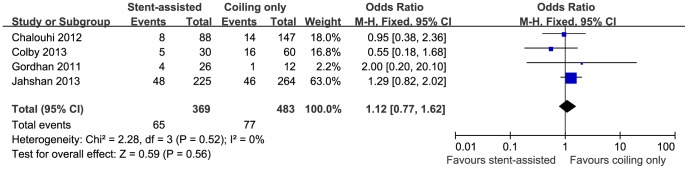
Forest plot of all-complication rate comparing stent-assisted coiling versus coiling only. Fixed-effect model was applied.

4 studies [9], [10], [11], [13] including a total number of 852 patients assessed the all-complication rate (369 patients received stent-assisted coiling and 483 coiling only). A large study conducted by Piotin et al. [6] only summarized the permanent complication rate and was excluded from this analysis. Documented complications included thromboembolism, transient ischemic attack, recanalization, aneurysm rupture, coil protrusion, coil fracture, dissection and sent migration. The pooling data revealed no significant difference in the occurrence of complications during and after either intervention (17.6% versus 15.9%; OR, 1.12; 95% CI, 0.77–1.62; P = 0.56) (Figure 4) nor did it indicate any heterogeneity (I^2^ = 0%).

**Figure 5:**
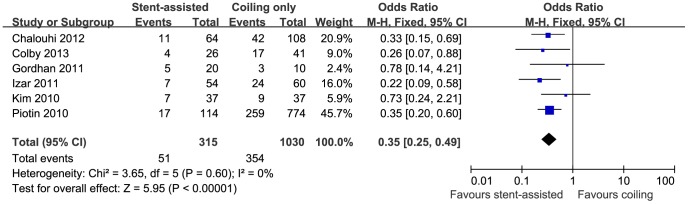
Forest plot of recurrence rate comparing stent-assisted coiling versus coiling only. Fixed-effect model was applied.

6 studies [6], [9], [10], [11], [12], [14] with a total of 1345 patients evaluated recurrence rate via angiography during follow up (315 patients received stent-assisted coiling and 1030 coiling only). The stent-assisted coiling significantly reduced the recurrence rate compared with embolization via coiling alone (16.2% versus 34.4%; OR, 0.35; 95% CI, 0.25–0.49; P<0.00001) (Figure 5). The recurrence rate demonstrated no heterogeneity (I^2^ = 0%).

### Analysis of secondary outcomes

Immediate packing density, mortality rate, permanent complication rate and thromboembolic complication rate were classified as secondary outcomes. 3 studies [6], [11], [12] (1474 patients) revealed a mean packing density of 27.4% in the stent-assisted coiling group compared to 28.2% in the coiling only group. 3 studies [6], [8], [15] (1473 patients) that reported mortality rate revealed no significant difference between the stent-assisted coiling and coiling only groups (9.1% versus 2.6%; OR, 2.31; 95% CI, 0.68–7.82; P = 0.18) (Figure S2). In particular, patients recruited in the study by Kung et al. [15] were all diagnosed with subarachnoid aneurysmal hemorrhage. Pooling the data of 4 studies [6], [8], [9], [13] (1123 patients) concerning permanent complications revealed no significant difference between the two groups (5.6% versus 3.9%; OR, 1.52; 95% CI, 0.96–2.41; P = 0.08) (Figure S3). Analysis on thromboembolic complications of 4studies [9], [10], [11], [16] (457 patients) did find one intervention better than the other (4.2% versus 4.9%; OR, 0.99; 95% CI, 0.41–2.38; P = 0.97) (Figure S4).

### Sensitivity analysis and publication bias

Funnel plot analysis on all outcomes are shown in Figure S5, which indicated significant publication bias in immediate occlusion rate and mortality rate.

## Discussion

Until now, this is the first meta-analysis comparing the two most popular interventional techniques in the treatment of intracranial aneurysms. The results of this meta-analysis show that stent-assisted coiling significantly reduces the recurrence rate while not increasing the occurrence of procedural related complications compared with coiling only technique. Moreover, although the delivery of the stent tends to reduce the immediate occlusion rate and initial packing density, it promotes the thrombosis within the aneurysm, which may be linked to the lower recurrence rate [6], [9], [22], [23].

The results of this meta-analysis should be cautiously interpreted. The data were all pooled from observational studies since no randomized control studies are available yet. As a result, the baseline characteristics vary among the included studies. Colby et al. and Ogilvy et al. focused on aneurysms located in the paraclinoid region [10], [16], while Chalouhi et al. studied patients with basilar tip aneurysms [9]. Kung et al. recruited a subset of patients with acutely ruptured aneurysms [15], while Gordhan et al. compared different interventions on non-ruptured aneurysms [11]. The results of this meta-analysis, however, are meaningful in a broad sense, but further subgroup analysis is still necessary to compare the effectiveness and safety of the two treatment modalities in aneurysms with different locations and status.

Since in most studies the treatment strategy used for each patient was largely based on the personal experience of the clinician, intergroup mismatch could not be avoided. As summarized in table 1, the aneurysm size and status were the most prominent discriminations between the two groups. A well-designed randomized study would be ideal in eliminating these biases. Such studies, however, are difficult to be carried out since large and wide-neck aneurysms are not suitable candidates for traditional coiling embolization [3], [4], [24] and the administration of dual anti-platelet treatment hinders the application of stent in subarachnoid aneurysmal hemorrhage [15], [18], [25]. Instead, a well-designed prospective cohort study could be a more feasible approach.

Our study found that compared with the coiling only technique, aneurysms treated with stent-assisted coiling tend to advance through continuous thrombosis towards a more complete occlusion after the operation. The advantage of stent-assisted coiling over coiling only in terms of occlusion rate was not prominent on the immediate angiography after the surgery. Jahshan et al. and Piotin et al. obtained significantly lower initial occlusion rate by stent-assisted coiling [6], [13]. The result from pooling data leaned insignificantly towards a higher occlusion rate without stent placement (57.6% versus 68.7%; OR, 0.66; 95% CI, 0.30–1.44; P = 0.30) (Figure 2), which correlated with a trivial higher packing density in the coiling only group. Yet angiography several months later showed significantly more progressive thrombosis by stent-assisted coiling (37.5% versus 19.4%; OR, 2.75; 95% CI, 1.95–3.86; P<0.00001) (Figure 3). In the study by Jahshan et al., the total occlusion rate dropped from 89.4% to 48.9% in the coiling only group, while that of the stent-assisted coiling group increased from 59.6% to 62.7% during follow up [13]. Another study by Fiorella et al. determined a progressive thrombosis rate of 52% by stent-assisted coiling during a median follow up of 4.6 months [23]. A meta-analysis showed that approximately 45% of aneurysms were completely occluded by stent-assisted coiling at immediate angiography and this rate rose to 61% during follow up [17]. The less initial package dense possibly results from the difficulty in manipulating coil catheter with stent implanted at first place [6]. Due to the flow diversion effect and facilitation of endotheliazation by stent, the stent-assisted coiling might induce changes in intra-aneurysmal hemodynamics that are thought to promote delayed thrombosis and offer more stable occlusion in the long-term [10], [22].

In agreement with other studies [26], [27], this meta-analysis strongly supported that aneurysms treated with stent-assisted coiling are less prone to recurrence (Figure 5). Data from coiling embolization demonstrated that incomplete and loose packing are the direct and primary causes for aneurysmal recurrence [28]. Other studies by Kawanabe et al. and Sluzewski et al., have showed that packing densities of at least 20 to 25% are needed to protect against recurrence [29], [30]. Although the stent-assisted coiling does not have advantage over coiling in terms of initial packing density according to our data, progressive thrombosis and the better arterial wall reconstruction might lead to the better outcome [6], [9], [22], [23]. Moreover, stent-assisted coiling has also been shown to significantly reduce the intra-aneurysmal flow velocity, which might be another contributing factor [31]. However, the follow up duration of stented aneurysm, which also influences the recurrence rate, are shorter in four out of six studies, as displayed in table 1. This is an inevitable drawback of observational study since most of the stents were implanted during the recent years. On the other hand, the larger aneurysm size in the stent-assisted coiling group, which is also a risk factor for aneurysmal recurrence, confers more robust advantage of stent assistance technique in reducing recurrence.

The all-complication rate (Figure 4), permanent complication rate (Figure S3) and mortality rate (Figure S2) of stent-assisted coiling were comparable to the conventional coiling embolization, implying its relatively safe application in the treatment of complex aneurysms. The study by Piotin et al. showed significantly higher permanent complication rate in stent-assisted coiling group [6], but the results were distorted because of the inclusion of balloon-expandable stents, which were responsible for increased morbidity, and the error introduced by imbalanced subjects between two groups. When it comes to the ruptured aneurysm, a systematic review demonstrated only 81% had good clinical outcome after stent-assisted coiling treatment of aneurysms with Hunt-Hess score I-III, which was unfavorable compared with data for conventional coiling without a stent [18].

However, for ruptured wide-neck intracranial aneurysms Tahtinen et al. found 69% had a good clinical outcome, which was superior to that of conventional coiling, and the authors suggested that stent-assisted coiling could also be a possibility in treating SAH with complex morphology [32]. Since none of these studies had control groups, controlled studies are needed to generate more robust conclusions in guiding the appropriate treatment choice (stent-assisted coiling or coiling only) of ruptured aneurysms.

For unruptured aneurysm, dual antiplatelet therapy is routinely administrated to prevent the occurrence of thrombotic events [9], [33], [34]. Traditional remedy includes aspirin and clopidogrel. Unfortunately, Prabhakaran et al. found that clopidogrel resistance occurred in half of the patients undergoing cerebrovascular stent placement [35], and Reavey-Cantwell et al. showed that 21% of patients receiving endovascular neurological procedures were nonresponders to aspirin [36]. Thromboembolic events were also shown to be associated with anti-platelet resistance [37], [38]. Preoperational screening for the nonresponders, as well as the use of other effective drugs, such as prasugrel or ticlopidine, were applied by Chalouhi et al. and Jahshan et al. aiming to decrease the thrombotic events [9], [13]. The result from our meta-analysis, showed that the stent-assisted coiling did not have a tendency towards higher thromboembolic complication, largely influenced by the new treatment strategy of Chalouhi which accounted for nearly 70% (Figure S4). Research exploring the safety of the new therapy showed that aspirin/prasugrel may predispose to a higher risk of hemorrhage during neurointerventional surgery compared with aspirin/clopidogrel (19.4 vs. 3.6%, P = 0.02) [25]. Therefore the safety and efficacy of new anti-platelet therapy needs to be further evaluated.

## Conclusion

Here we are the first to present the Meta-analysis comparing the stent-assisted coiling and coiling only techniques in treatment of intracranial aneurysms. Although heterogeneity exists among the recruited reports, current published data suggests that aneurysms treated with stent-assisted coiling has a lower recurrence rate, possibly due to a continuous thrombosis process towards a more complete occlusion after the operation. Furthermore, there is no significant difference in terms of all-complication rate, permanent complication rate, mortality rate and thromboembolic events between the stent-assisted coiling and the conventional coiling techniques. This study emphasizes the safety and effectiveness of stent-assisted techniques. Further validation by well-designed randomized or prospective cohort studies are still needed.

## Supporting Information

Figure S1 **Forest plot of immediate occlusion rate comparing stent-assisted coiling versus coiling only**. Fixed-effect model was applied.
(TIF)

Figure S2 **Forest plot of mortality rate comparing stent-assisted coiling versus coiling only**. Random-effect model was applied.
(TIF)

Figure S3 **Forest plot of permanent complication rate comparing stent-assisted coiling versus coiling only.** Fixed-effect model was applied.
(TIF)

Figure S4 **Forest plot of thromboembolic complication rate comparing stent-assisted coiling versus coiling only.** Fixed-effect model was applied.
(TIF)

Figure S5 **Funnel plots**. Immediate occlusion rate (A), progressive thrombosis (B), all-complication rate (C), recurrence rate (D), mortality rate (E), permanent complication rate (F), thromboembolic complication rate (G).
(TIF)

Checklist S1 **The PRISMA 2009 Checklist.**
(DOC)
