# Supplementary figures and images for: Stent-Assisted Coiling versus Coiling in Treatment of Intracranial Aneurysm: A Systematic Review and Meta-Analysis
Source: PLoS One. 2014 Jan 15;9(1):e82311. doi: 10.1371/journal.pone.0082311 (PMC3893071; doi:10.1371/journal.pone.0082311)

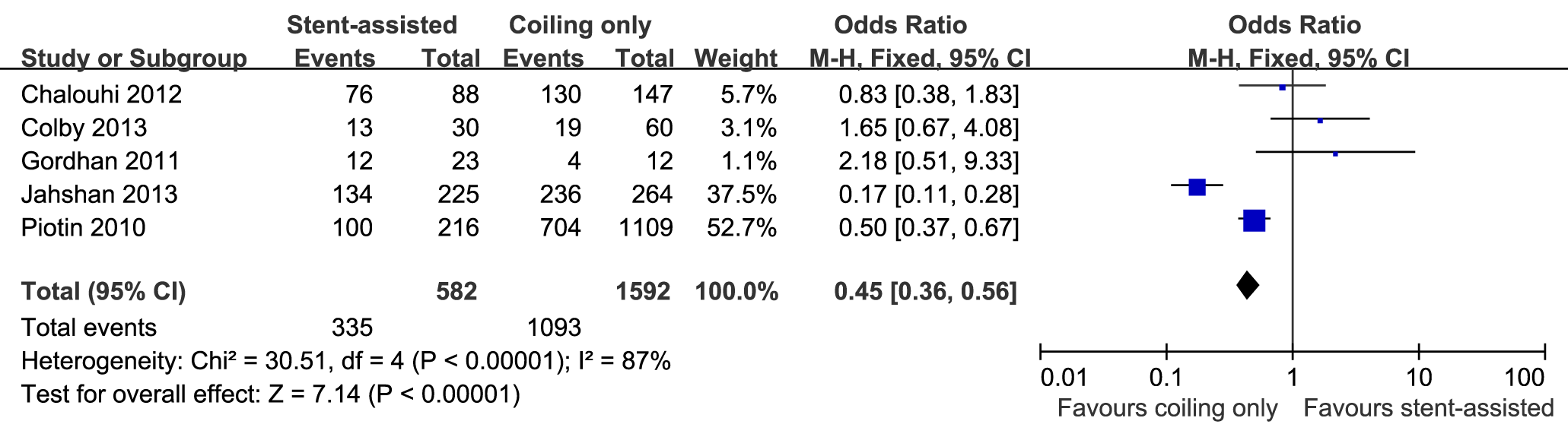

Supplement: Figure S1 — Forest plot of immediate occlusion rate comparing stent-assisted coiling versus coiling only. Fixed-effect model was applied. (TIF) [file pone.0082311.s001.tif]

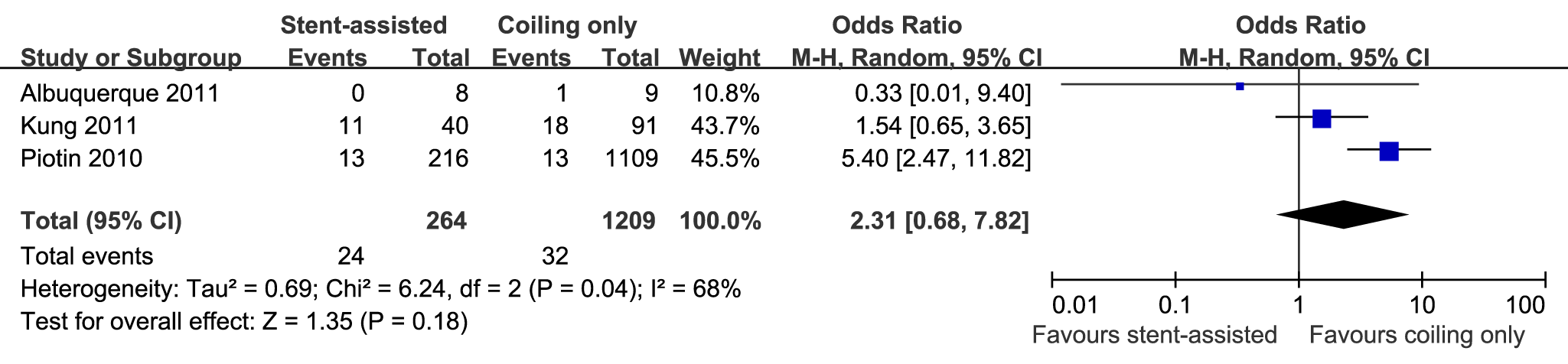

Supplement: Figure S2 — Forest plot of mortality rate comparing stent-assisted coiling versus coiling only. Random-effect model was applied. (TIF) [file pone.0082311.s002.tif]

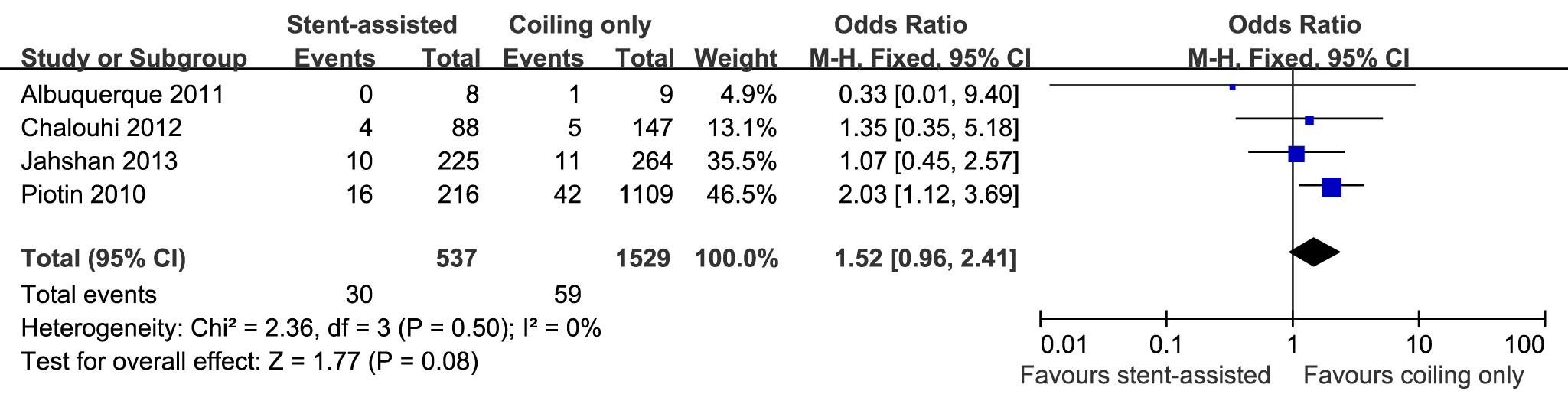

Supplement: Figure S3 — Forest plot of permanent complication rate comparing stent-assisted coiling versus coiling only. Fixed-effect model was applied. (TIF) [file pone.0082311.s003.tif]

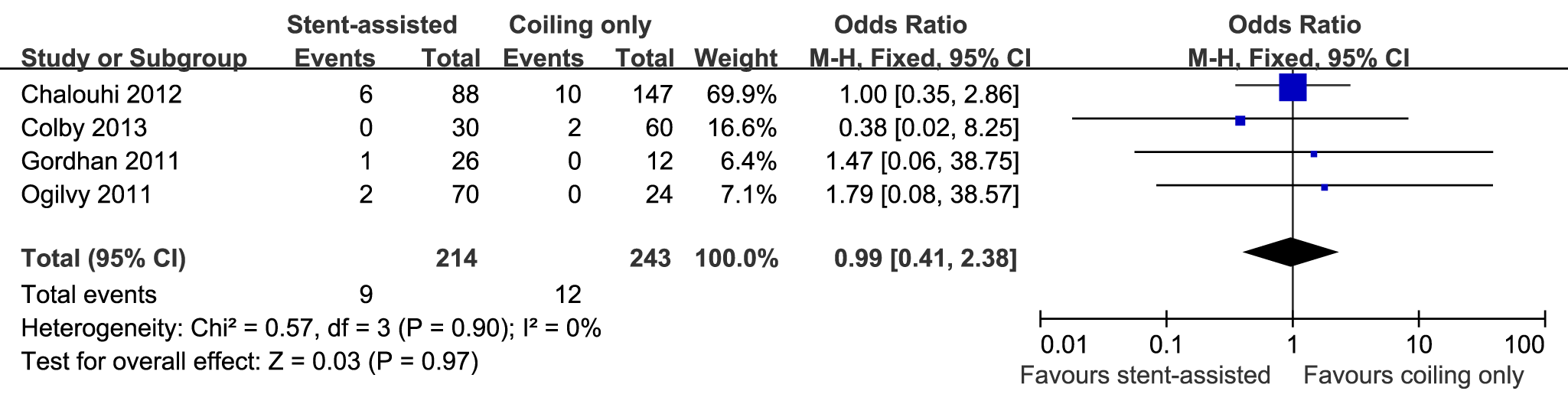

Supplement: Figure S4 — Forest plot of thromboembolic complication rate comparing stent-assisted coiling versus coiling only. Fixed-effect model was applied. (TIF) [file pone.0082311.s004.tif]

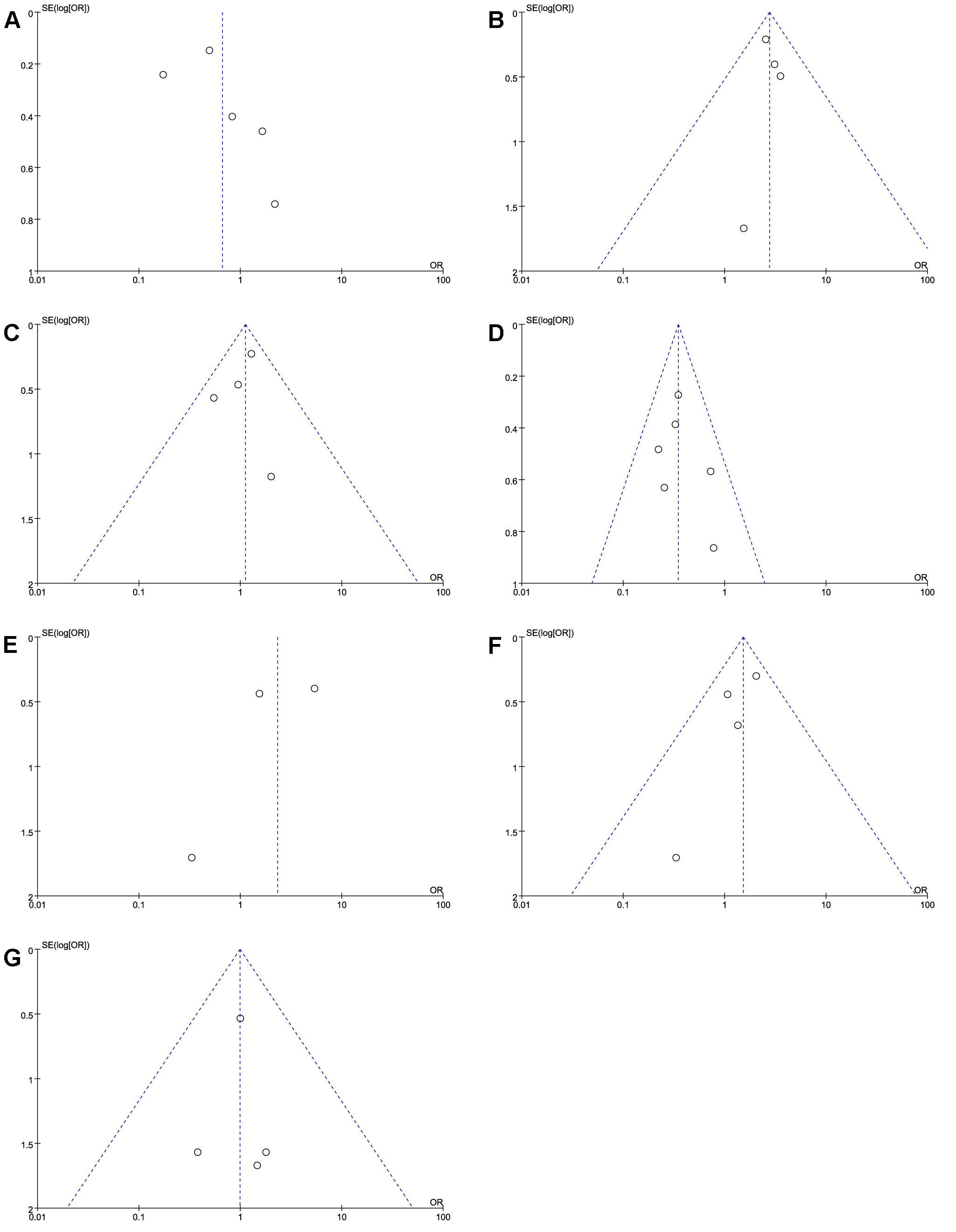

Supplement: Figure S5 — Funnel plots. Immediate occlusion rate (A), progressive thrombosis (B), all-complication rate (C), recurrence rate (D), mortality rate (E), permanent complication rate (F), thromboembolic complication rate (G). (TIF) [file pone.0082311.s005.tif]
